# Sustaining Interferon Induction by a High-Passage Atypical Porcine Reproductive and Respiratory Syndrome Virus Strain

**DOI:** 10.1038/srep36312

**Published:** 2016-11-02

**Authors:** Zexu Ma, Ying Yu, Yueqiang Xiao, Tanja Opriessnig, Rong Wang, Liping Yang, Yuchen Nan, Siba K. Samal, Patrick G. Halbur, Yan-Jin Zhang

**Affiliations:** 1 Molecular Virology Laboratory, VA-MD College of Veterinary Medicine, University of Maryland, College Park, MD, USA; 2 The Roslin Institute and The Royal (Dick) School of Veterinary Studies, University of Edinburgh, Roslin, Midlothian, UK; 3 Department of Veterinary Diagnostic and Production Animal Medicine, College of Veterinary Medicine, Iowa State University, Ames, IA, USA; 4 Virology Laboratory, VA-MD College of Veterinary Medicine, University of Maryland, College Park, MD, USA

## Abstract

Porcine reproductive and respiratory syndrome virus (PRRSV) strain A2MC2 induces type I interferons in cultured cells. The objective of this study was to attenuate this strain by serial passaging in MARC-145 cells and assess its virulence and immunogenicity in pigs. The A2MC2 serially passaged 90 times (A2MC2-P90) retains the feature of interferon induction. The A2MC2-P90 replicates faster with a higher virus yield than wild type A2MC2 virus. Infection of primary pulmonary alveolar macrophages (PAMs) also induces interferons. Sequence analysis showed that the A2MC2-P90 has genomic nucleic acid identity of 99.8% to the wild type but has a deletion of 543 nucleotides in nsp2. The deletion occurred in passage 60. The A2MC2-P90 genome has a total of 35 nucleotide variations from the wild type, leading to 26 amino acid differences. Inoculation of three-week-old piglets showed that A2MC2-P90 is avirulent and elicits immune response. Compared with Ingelvac PRRS^®^ MLV strain, A2MC2-P90 elicits higher virus neutralizing antibodies. The attenuated IFN-inducing A2MC2-P90 should be useful for development of an improved PRRSV vaccine.

Porcine reproductive and respiratory syndrome (PRRS) is an economically important swine contagious disease across the world, which has resulted in an estimated $664 million loss per year to the swine industry in the United States alone^1^. The causative agent of the contagious disease is PRRS virus (PRRSV), a positive-sense single-stranded RNA virus of the family *Arteriviridae*^2^,^3^. The main target cells for PRRSV infection of pigs are pulmonary alveolar macrophages (PAMs)^4^. PRRSV propagation *in vitro* is generally conducted in MARC-145 cells, derived from MA-104, a kidney cell line of an African green monkey^5^.

PRRSV appears to inhibit synthesis of type I interferons (IFNs) in pigs, whereas swine transmissible gastroenteritis virus (TGEV) and porcine respiratory coronavirus (PRCV) induce high levels of IFN-α^6^,^7^,^8^. PRRSV antagonizes induction of type I IFNs in both PAMs and MARC-145 cells as infection of the cells *in vitro* leads to very low level interferon-α (IFN-α) expression^6^,^9^,^10^. Type I IFNs are critical to the innate immunity against viral infections and play an important role in activation of the adaptive immune response^11^,^12^. Adenovirus-mediated expression of IFN-α in pigs leads to reduction in disease signs when the animals were challenged with PRRSV^13^. Presence of the exogenous IFN-α at the time of PRRSV infection alters innate and adaptive immune responses by increasing IFN-γ secreting cells and changing cytokine profile in the lung 14 days post-infection^14^.

An atypical type 2 PRRSV strain A2MC2 induces synthesis of type I IFNs in the cultured cells and replication of A2MC2 is needed for the IFN induction, whereas PRRSV strains VR-2332, Ingelvac PRRS^®^ MLV, NVSL 97–7895 and VR-2385 do not induce detectable IFNs^15^. Experimental infection of pigs with the A2MC2 strain leads to earlier onset and higher levels of virus-neutralizing antibodies than the Ingelvac PRRS^®^ MLV vaccine strain^16^. Virus neutralizing antibodies against PRRSV confer protection of pigs against challenge with virulent strain^17^. Passive transfer of PRRSV-neutralizing antibodies in pregnant sows confers sterilizing immunity against reproductive failure induced by virulent strain challenge. Passive transfer with PRRSV-neutralizing antibodies to young weaned pigs blocks PRRSV viremia from challenge^18^.

Despite substantial efforts to control PRRS, no production or vaccination regimen has demonstrated sustaining success^19^,^20^. This is likely in part due to biosecurity challenges and both antigenic and genomic variations among PRRSV isolates, allowing for frequent transmission between pig populations and persistence of the virus in infected pigs^21^. Attenuated live virus vaccines have been commercially available for over two decades, however, PRRS remains one of the top challenges for swine producers and outbreaks of PRRS. Therefore, an improved vaccine is needed to prevent and control PRRS.

In the present study, the objective was to attenuate the A2MC2 strain in MARC-145 cells by serial passaging and assess virulence and immunogenicity of the high passages, as previously moderate virulence of the wild type strain was observed in pigs^16^. Interestingly, the feature of IFN induction of the A2MC2 strain is sustained during the serial passaging, as passage 90 of the virus is still capable to induce interferon synthesis. A multi-step growth assay of high-passaged A2MC2-P90 virus showed that it propagates faster than the wild type virus with a higher virus yield. A pig study indicates that the A2MC2-P90 is avirulent and elicits higher virus-neutralizing antibodies than Ingelvac PRRS^®^ MLV vaccine strain. Overall, these results demonstrated that the avirulent A2MC2-P90 virus retains the feature of IFN induction and should be useful as a candidate for development of an improved vaccine against PRRS.

## Materials and Methods

### Cells and viruses

MARC-145^5^ and Vero (ATCC CCL-81) cells were grown in Dulbecco’s Modified Eagle Medium (DMEM) supplemented with 10% fetal bovine serum (FBS). CRL-2843 (porcine macrophages, ATCC) were cultured in RPMI1640 medium supplemented with 10% FBS. Primary PAM cells were prepared from 4-8-week-old piglets and cultured in RPMI1640 medium supplemented with 10% FBS^22^.

PRRSV strain A2MC2, VR-2385 and Ingelvac PRRS^®^ MLV were propagated and titrated in MARC-145 cells. Virus yields were titrated by 10-fold serial dilutions and presented as the median tissue culture infectious dose (TCID_50_)^23^. Newcastle disease virus (NDV) strain LaSota carrying the gene of green fluorescence protein (NDV-GFP) was propagated and titrated in Vero cells^24^.

### Interferon bioassay

Detection of presence of IFNs in culture supernatant from PRRSV-infected MARC-145 cells was done as previously described^15^. Briefly, the supernatant was diluted in DMEM and used to treat Vero cells in 96-well plates overnight, followed by inoculation with NDV-GFP. Fluorescence microscopy was conducted 24 h after NDV inoculation to observe GFP-positive cells.

### Immunofluorescence assay (IFA)

PRRSV propagation in MARC-145 cells was detected with IFA using an N-specific monoclonal antibody EF11^25^. The infected cells in 96-well plate were fixed and rinsed with phosphate-buffered saline (PBS) pH7.2 before addition of the EF11 antibody. DyLight™ 488 conjugated goat anti-mouse IgG (Rockland Immunochemicals Inc., Limerick, PA) was used to detect the EF11 binding to the N protein in the infected cells. Observation of N-positive cells was conducted under fluorescence microscopy.

### Western blotting

Total proteins in cell lysate samples were separated by sodium dodecyl sulfate-polyacrylamide gel electrophoresis (SDS-PAGE), followed by transfer to nitrocellulose membrane^26^. Blotting of the membrane with antibodies against RIG-I (Santa Cruz Biotechnology, Inc., Dallas, TX) and tubulin (Sigma-Aldrich Corp, St. Louis, MO) was conducted. Horseradish peroxidase-conjugated secondary antibodies (Rockland Immunochemicals Inc.) and chemiluminescence substrate were used to reveal specific reactions by the primary antibodies. Chemi-Doc Imaging System (Bio-Rad, Hercules, CA) was used to capture the luminescence signal.

### RNA isolation, reverse transcription, and real-time PCR

Total RNA was isolated with the TRIzol Reagent (Life Technologies Corporation, Carlsbad, CA) following the manufacturer’s instructions. Reverse transcription followed by PCR (RT-PCR) and real-time PCR were conducted to amplify target PRRSV sequences or to determine PRRSV RNA level^22^,^27^. Detection of ribosomal protein L32 (RPL32) expression in the same sample was conducted to normalize the total input RNA. Primers of real-time PCR in this study were previously described^28^ and analysis of relative transcript levels was performed by normalization of RPL32 in comparison with controls.

For RT-PCR to determine possible deletions in the nsp2 region of the A2MC2 genome during high passages, primers 85nspF3 (5′CTCGACGAACTCAAAGACC3′) and 32nsp2R2 (5′CTGCGGACGGAGCTGATGTGC3′) were used to amplify the target fragment with Phusion Flash High-Fidelity PCR Master Mix (Thermo Fisher Scientific, Pittsburgh, PA).

### Plaque assay

A plaque assay in MARC-145 cells was done to compare the growth property of A2MC2 high passage with the wild type virus^15^. Briefly, PRRSV A2MC2 was diluted to 10 and 100 TCID_50_ per ml and added to the monolayer cells in 6-well plates at 1 ml per well. After 2 h incubation at 37 °C, the inoculum was removed and 3 ml 0.5% agarose overlay containing the complete growth medium was added. The cells were stained at 72 h after incubation by addition of 2 ml neutral red mixture with agarose and observed for plaques after further overnight incubation.

### Virus neutralization assay

Virus neutralization assay was performed on MARC-145 cells to determine PRRSV-neutralizing antibodies in pig serum samples^16^. VR-2332, the prototype of type 2 PRRSV with nucleic acid identity of 99.8% to A2MC2^15^, was used as target virus in the assay at 100 TCID_50_ for each reaction. The starting dilution of serum samples was 1:8, followed by 2-fold serial dilutions. IFA with N-specific monoclonal antibody EF11 was conducted 24 h after inoculation of the cells. Compared to serum samples from mock-infected pigs, the reciprocal of the highest serum dilution that reduced 50% PRRSV replication was counted as the VN titer.

### Sequencing

RNA isolation from A2MC2 virions was done for reverse transcription, PCR amplification and DNA sequencing by chain-termination method using ABI Genetic Analyzer 3130 (ThermoFisher Scientific, Waltham, MA)^15^. Sequence assembly and analysis was done with LaserGene Core Suite (DNASTAR Inc., Madison, WI). The cDNA sequence of the full-length A2MC2-P90 genome has been deposited in to GenBank (accession number: KU318406).

### Animal study

Two animal studies were conducted after approval by Institutional Animal Care and Use Committees (IACUC) of the University of Maryland and Iowa State University according to relevant guidelines and policies for the care and use of laboratory animals. The first animal study was to determine the virulence of the high passages of A2MC2 virus. Three-week-old PRRSV-negative piglets weighing from 3.2 to 7.5 kg were randomly divided into five groups with 4 pigs in each group. The piglets in groups 1 to 4 were inoculated with 1 ml of PRRSV strains A2MC2-P9, A2MC2-P75, A2MC2-P90, and Ingelvac PRRS^®^ MLV, respectively, at 5 × 10^5^ TCID_50_/ml via intranasal (I.N.) inoculation, while group 5 was mock-infected with PBS pH7.2. The I.N. inoculation was used as PRRSV transmits via respiratory route. The pigs in each group were euthanized on day 14 post infection (DPI14) by pentobarbital overdose (FATAL-PLUS, Vortech Pharmaceuticals, LTD. Dearborn, MI). Visible macroscopic lung lesions and histopathology were scored and recorded as previously described^29^,^30^. The level of interstitial pneumonia was scored ranging from 0 (absent) to 6 (severe diffuse interstitial pneumonia). Scoring of macroscopic and microscopic lung pathology was done in a treatment status-blinded fashion independently by two veterinary pathologists (TO, PGH). If results disagreed, they were combined and the average was used for further analysis.

The second animal study was conducted to assess the immunogenicity of high passages of A2MC2. Three-week-old PRRSV-negative piglets were randomly divided into four groups with 4 pigs in each group. The piglets in groups 1 to 4 were inoculated with 1 ml of PRRSV A2MC2-P9, A2MC2-P90, and Ingelvac PRRS^®^ MLV, respectively, at 5 × 10^5^ TCID_50_/ml via intramuscular (I.M.) inoculation, while group 4 was mock-infected with PBS pH7.2. The I.M. route is generally used for porcine vaccination. Blood samples were collected weekly. The pigs were euthanized on DPI48. To assess the antibody response against PRRSV in the pigs, serum samples of DPI35 was tested with a commercial PRRSV ELISA kit (IDEXX PRRS X3 Ab Test; IDEXX Inc., Westbrook, MA, USA) according to the manufacturer’s instructions. A sample-to-positive (S/P) ratio greater than 0.4 was considered positive.

### Statistical analysis

Differences between treatment samples and control were assessed by the Student *t*-test. Differences between two groups for VN antibody titers of individual pigs were analyzed using analysis of variance (ANOVA). A two-tailed *P*-value of less than 0.05 was considered significant.

## Results

### Serial passaging of A2MC2 in MARC-145 cells

**Figure 1:**
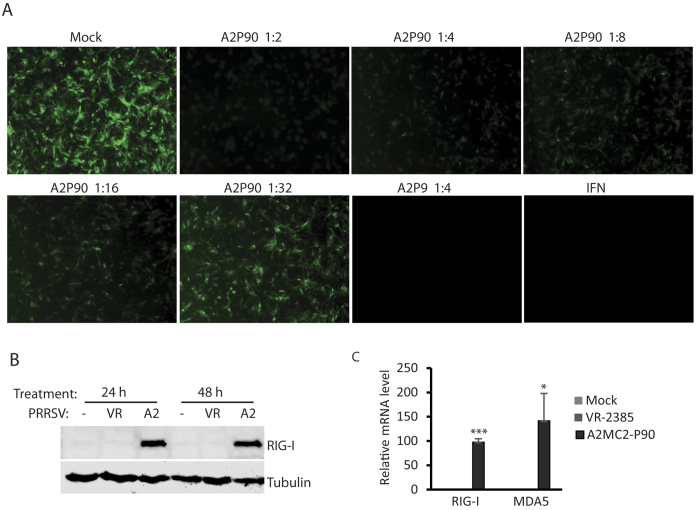
A2MC2-P90 induces synthesis of interferons. (**A**) Interferon bioassay in Vero cells. Dilutions of cell culture supernatant of MARC-145 cells infected with A2MC2-P90 (A2P90) or A2MC2-P9 (A2P9) were used to treat Vero cells. Treatment with 1000 U IFN-α was included as a control. At 12 h after the treatment, the Vero cells were inoculated with NDV-GFP. At 24 h post-inoculation of NDV, the cells were observed under fluorescence microscopy. (**B**) Elevation of RIG-I in Vero cells treated with A2MC2-P90 supernatant. The Vero cells were treated with culture supernatant from MARC-145 cells infected with VR-2385 (VR) or A2MC2-P90 (A2) and harvested 24 and 48 h post treatment for immunoblotting. (**C**) Treatment with A2MC2-P90 supernatant leads to elevation of RIG-I and MDA5 expression in Vero cells detected by real-time PCR. Relative levels of transcripts are shown in folds in comparison to treatment with supernatant from mock-infected MARC-145 cells. Significant difference from treatment with supernatant of mock-infected cells is denoted by “*”, which indicates *P* < 0.05 and “***” for *P* < 0.001.

PRRSV strain A2MC2 was subjected to serial passaging in MARC-145 cells to minimize previously observed moderate virulence^16^. The A2MC2 virus was passaged in MARC-145 cells for 90 consecutive passages. For each passage, the cells were frozen and thawed three times when cytopathic effect (CPE) occurred over 50% of the cells. Virus samples were collected for each passage. IFN bioassay results showed that treatment of Vero cells with the supernatnat of A2MC2 passage 90 (A2MC2-P90) even at a dilution of 1 to 16 inhibited the replication of NDV-GFP (Fig. 1A). This suggested that A2MC2-P90 retains the capacity of IFN induction of the wild type A2MC2.

To confirm IFN induction by strain A2MC2-P90, we performed immunoblotting detection of RIG-I, which is upregulated by type I IFNs^31^. Result showed that the RIG-I level increased in the Vero cells treated with culture supernatant from A2MC2-P90-infected MARC-145 cells, whereas no change in RIG-I level was observed in cells treated with supernatant from VR-2385-infected cells (Fig. 1B). Compared with treatment of mock-infected cells, the treatment with supernatant from A2MC2-P90 infected cells led to 97.8 and 141.5-fold higher RIG-I and MDA5 transcript levels, respectively, whereas treatment with supernatant from VR-2385-infected cells had only 0.9 and 0.7-fold of RIG-I and MDA5 transcript levels, respectively (Fig. 1C).

### Growth property determination and plaque assay

**Figure 2:**
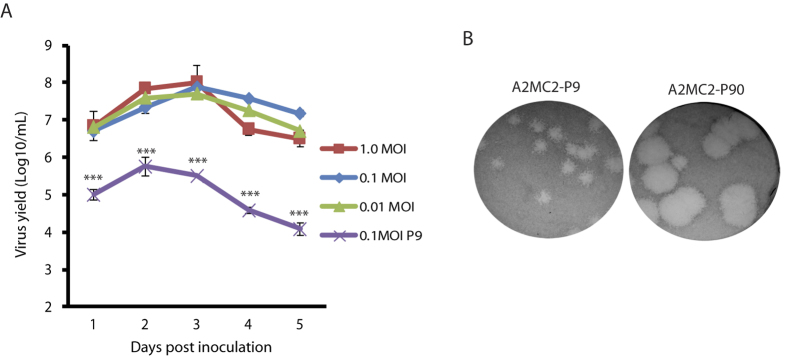
Growth properties of A2MC2-P90 in MARC-145 cells. (**A**) Multi-step growth curve of A2MC2-P90 in MARC-145 cells. The cells were inoculated with A2MC2 virus at a multiplicity of infection (MOI) of 0.01, 0.1 or 1 per cell. Inoculation of A2MC2-P9 at an MOI of 0.1 was included as a control. Virus yields at different time points after inoculation were titrated by an immunofluorescence assay. Error bars represent variation of three repeated experiments. “***”Denotes significant difference (*P* < 0.001) in virus yields between A2MC2-P90 and A2MC2-P9. (**B**) Plaque assay in MARC-145 cells. The cells were infected with diluted A2MC2-P9 and A2MC2-P90 and overlaid with agarose. Plaques were observed 4 days post-infection and photographed for comparison.

A2MC2-P90 was tested in MARC-145 cells for growth properties, including a multi-step growth curve and a plaque assay as described previously^15^. The virus yields reached its peak at 72 hours post inoculation (hpi) and were 7.7, 7.9 and 8.0 Log_10_/ml in TCID_50_ for the cells with inoculum at an MOI (multiplicity of infection) of 0.01, 0.1 and 1, respectively (Fig. 2A). The virus yields for the cells inoculated with A2MC2-P90 at an MOI of 0.1 and harvested at 24, 48, 72, 96 and 120 hpi were 6.7, 7.3, 7.8, 7.6 and 7.2 Log_10_/ml, respectively, which were significantly higher than the yields from the cells inoculated with wild type A2MC2 at an MOI of 0.1: 5.0, 5.7, 5.5, 4.6 and 4.1, respectively. Similar trends and titers of virus yields were observed for the samples harvested from the cells with the three different amounts of A2MC2-P90 inoculation.

A plaque assay was done for A2MC2-P90 and compared with wild type A2MC2. The plaque sizes of A2MC2-P90 were 8–10 mm in diameter, much bigger than the wild type A2MC2 palques, which were generally 3–4 mm in diameter (Fig. 2B). The larger size of plaques produced by A2MC2-P90 is consistent with its higher yield compared to the wild type virus.

### Sequencing of cDNA of A2MC2-P90 genomic RNA

**Figure 3:**
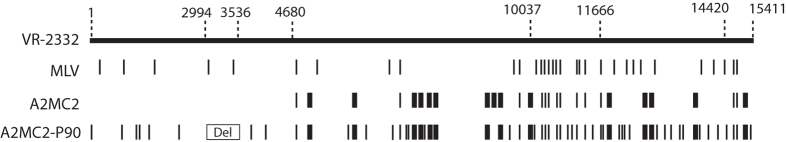
Illustration of sequence variation of A2MC2-P90 (GenBank accession number: KU318406) in comparison to VR-2332 (GenBank accession number: U87392), MLV (GenBank accession number: AF066183) and wild type A2MC2 (GenBank accession number: JQ087873). The top line indicates the genomic sequence of VR-2332 and the numbers above the line indicate nucleotide positions in the genome. The nucleotide variations in comparison with VR-2332 are indicated by vertical bars. The wide bars indicate the unique nucleotides identified earlier for A2MC2 in comparison with VR-2332 and MLV. A deletion (Del) in A2MC2-P90 from nt2994 to 3536 is indicated. For a list of non-synonymous nucleotide variations in A2MC2-P90 genome compared to wild type A2MC2, please refer to Table 1. For a full list of all nucleotide variations in A2MC2-P90 genome compared to wild type A2MC2, VR-2332 and Ingelvac PRRS^®^ MLV, please refer to Supplemental Table 1.

The virions of A2MC2-P90 were used for RNA isolation and RT-PCR. DNA sequencing of the PCR products was done and compared with sequences of wild type A2MC2 virus. Variations of nucleotides and derived amino acids in comparison with wild type A2MC2, VR-2332 and Ingelvac PRRS^®^ MLV were identified (Supplemental Table 1). The locations of the differences in genomic RNA are illustrated in Fig. 3. The A2MC2-P90 genome has a deletion of 543 nucleotides (2994–3536) in ORF1a in comparison with wild type A2MC2 virus, leading to a deletion of 181 amino acid residues in hypervariable region of nsp2. Moreover, compared to the wild type, the A2MC2-P90 has 35 nucleotide mutations, among which 26 are non-synonymous, leading to 26 amino acid changes (Table 1).

Interestingly, among the 15 unique nucleotides in A2MC2 genome compared with Ingelvac PRRS^®^ MLV and VR-2332^15^, 14 remained the same in the A2MC2-P90 genome (Fig. 3). As a result, 5 of the 6 unique amino acid residues of A2MC2 compared to the MLV and VR-2332 remained the same in A2MC2-P90. The conserved five nucleotides leading to unique amino acids in A2MC2 are nt7621, 9655, 12012, 12972 and 12975 and the five unique residues are Ser20 in nsp8/9, Leu13 in nsp10, Gly135 in nsp12, and Val93 and Val94 in GP3. This result indicates that the 14 nucleotides in A2MC2 are highly conserved and sustained during the 90 serial passages. It also suggests that these 14 nucleotides or their related RNA structures might correlate with the feature of A2MC2 in IFN induction.

### The deletion in ORF1a occurs in passage 60 of A2MC2

**Figure 4:**
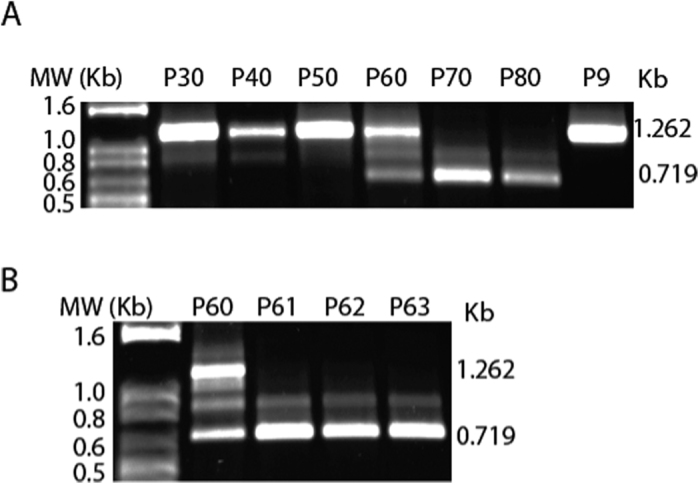
Identification of the initial A2MC2 passage that carries the nsp2 deletion in genome. (**A**) PCR detection of the nsp2 deletion in A2MC2 passages. The passages P30 to P80 were tested. Wild type A2MC2 (P9) was included as a control. The PCR product from the genome without deletion is 1.262 kb and it is 0.719 kb from the genome with the nsp2 deletion. (**B**) Identification of the initial A2MC2 passage that has the nsp2 deletion. The passages P60 to P63 were tested.

Having noticed the deletion in nsp2 of A2MC2-P90, we wondered at which passage the deletion occurred. RT-PCR was conducted to amplify a fragment spanning the deletion area. The expected sizes of the PCR product are 719 bp for A2MC2-P90 and 1262 bp for wild type A2MC2. The PCR products for passage 30, 40 and 50 are the same size as wild type A2MC2, while the sizes of passage 70 and 80 are the same as A2MC2-P90 (Fig. 4A). There were two main bands in PCR products of passage 60. Therefore, the deletion likely occurred around passage 60. PCR amplification of passage 60 through 63 showed that the size shift from 1262 bp to 719 bp likely occurred from passage 60 to 61 (Fig. 4B). The size shift suggests that mutant virus with the spontaneous deletion appeared to become the main virus quickly.

### A2MC2-P90 induces interferons in PAM cells

**Figure 5:**
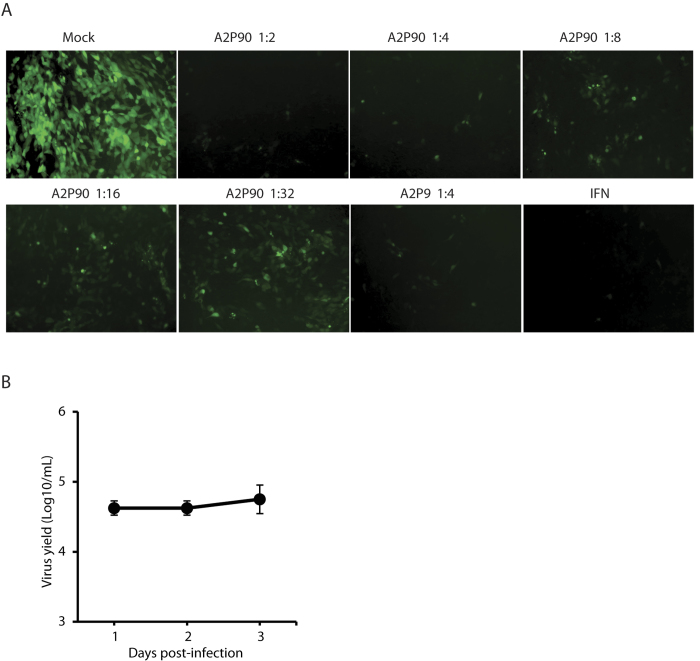
A2MC2-P90 induces IFN synthesis in PAM cells. (**A**) IFN bioassay in CRL2843 cells, in comparison with wild type A2MC2. IFN-α was included as a positive control. (**B**) Multi-step growth curve in PAM cells. The cells were inoculated with A2MC2-P90 at an MOI of 0.5. Virus yields were titrated on MARC-145 cells. Error bars represent variation of three repeated experiments.

PAMs are the major target cells for PRRSV infection *in vivo*^4^. To determine if A2MC2-P90 can infect PAMs and induce interferons, we inoculated PAMs with the high-passage virus at an MOI of 3. Wild type A2MC2 was included as a control. Interferon bioassay was conducted on CRL-2843 cells, immortalized porcine macrophages that are not susceptible to PRRSV, as reported^15^. Results showed that the supernatant of the A2MC2-P90 infected PAMs induced an antiviral effect in CRL-2843 cells by blocking the replication of NDV-GFP (Fig. 5A). The supernatant dilutions at 1 to 32 still induced inhibition of NDV-GFP.

A multi-step growth curve was also done to determine the propagation of A2MC2-P90 in PAM cells. The virus yields of PAMs inoculated at an MOI of 0.5 were 4.6, 4.6 and 4.8 Log10/ml 24, 48 and 72 hpi, respectively (Fig. 5B). A2MC2-P90 appears to be able to replicate in the primary cells though at low level.

### Non-virulence of A2MC2-P90 *in vivo*

**Figure 6:**
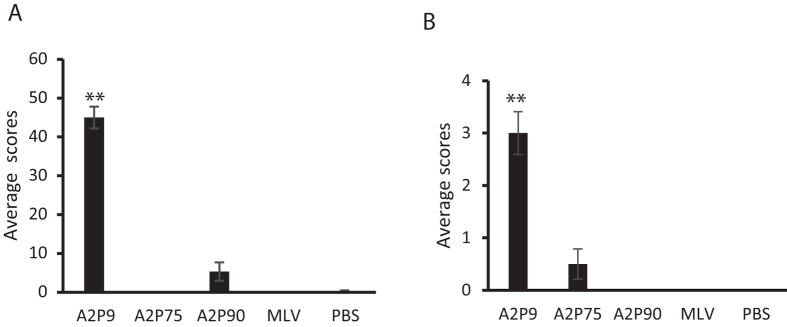
Lung lesions in pigs infected with PRRSV A2MC2-P9, A2MC2-P75, A2MC2-P90 and MLV at 14 days post-infection. Four pigs from each group were necropsied. Mock-infected pigs (PBS) were included as controls. (**A**) Average macroscopic lung lesion scores. Error bars represent standard errors of the scores among the four pigs in each group. A2: A2MC2. (**B**) Average microscopic lung lesion scores. Significant differences between the group of A2MC2-P9-infected pigs and each of the rest groups are denoted by “**”, which indicates *P* < 0.01.

The objective of the serial passaging of A2MC2 was to attenuate the strain. To determine the degree of attenuation of the A2MC2-P90, we conducted an animal study by inoculating 3-week-old PRRSV-negative piglets. A2MC2-P9, A2MC2-P75, and Ingelvac PRRS^®^ MLV were included in the animal study for control. Compared with pigs inoculated with A2MC2-P9, the pigs infected with A2MC2-P75 and A2MC2-P90 had significantly lower macroscopic lung lesion scores, like the MLV-infected pigs and the mock-infected control in magnitude (Fig. 6A).

Microscopically, the interstitial pneumonia scores of the pigs infected with A2MC2-P75 and A2MC2-P90 were significantly lower than pigs infected with A2MC2-P9 (Fig. 6B). Both A2MC2-P75 and A2MC2-P90 groups had pathology scores similar to the MLV-infected or mock-infected pigs. All the groups except for A2MC2-P9 had no significant difference from the mock-infected control group. These results suggest that under the study conditions, A2MC2-P75 and A2MC2-P90 are avirulent in pigs, like the MLV strain.

### A2MC2-P90 elicits higher level virus-neutralizing antibodies than the MLV strain

**Figure 7:**
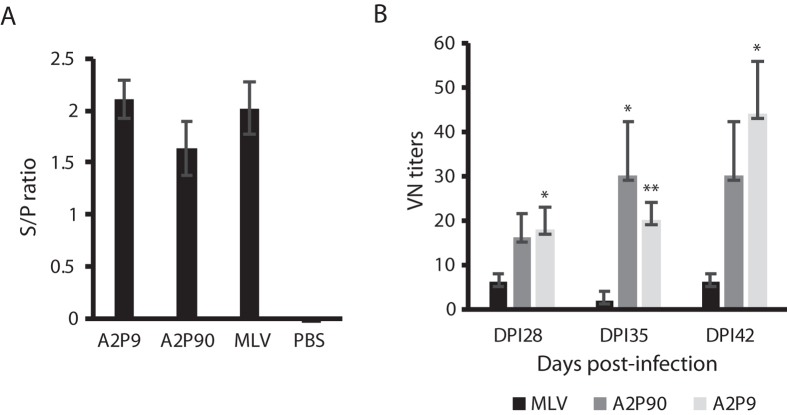
Serological testing of serum samples from pig studies. Four pigs from each group were infected. Mock-infected pigs (PBS) were included as controls. (**A**) ELISA of PRRSV antibodies in serum samples of 35 days post-infection (DPI). The S/P ratio above 0.4 is considered positive. Error bars represent standard errors of the ratios among the four pigs in each group. A2: A2MC2. (**B**) Virus-neutralization antibody assay against PRRSV VR-2332. The virus neutralization (VN) titers are shown as reciprocals of serum dilutions shown VN activity. Significant differences between the A2MC2 groups and the group of MLV-infected pigs are denoted by *and **, which indicate *P* < 0.05 and *P* < 0.01, respectively.

To assess the immunogenicity of A2MC2-P90, we conducted an animal study by inoculating 3-week-old PRRSV-negative piglets with A2MC2-P9, A2MC2-P90, and Ingelvac PRRS^®^ MLV viruses. ELISA result showed that all pigs that were inoculated with the PRRSV viruses developed specific antibodies by DPI35, whereas the pigs of mock-infected group were all negative (Fig. 7A). The average S/P ratios for the virus-infected groups were over 1.6 for all groups without significant difference.

Virus-neutralizing antibody assay was conducted for serum samples of DPI28 to DPI42 based on our previous study showing the appearance of VN antibody at DPI28^16^. All pigs in groups of A2MC2-P9 and A2MC2-P90, and three of four in MLV group had detectable VN antibodies at DPI28 (Fig. 7B). The average VN titers of the A2MC2-P90 and A2MC2-P9 groups were higher than those in MLV group for DPI28, DPI35 and DPI42 samples. The results show that similar to the wild type A2MC2 virus, A2MC2-P90 elicits higher VN antibodies than the MLV strain in this study.

## Discussion

Interferon induction is a unique characteristic of PRRSV strain A2MC2 as PRRSV strains generally antagonize interferon synthesis^8^,^15^. Considering the importance of interferons in activating the adaptive immune response, this feature may be desired in vaccine development against PRRS. Remarkably, the capability of strain A2MC2 to induce interferons is sustained after 90 serial passages in MARC-145 cells. Like the wild type virus, the high-passage virus also induces interferons in PAM cells. Moreover, the A2MC2-P90 is attenuated shown by its non-virulence in pigs and elicits higher virus-neutralizing antibodies.

Sequence comparison showed that 14 of 15 unique nucleotides of A2MC2 in comparison with both VR-2332 and MLV^15^ are conserved in the A2MC2-P90 genome. Among the six unique amino acid residues, five are identical in both wild type A2MC2 and A2MC2-P90 and are located in nsp8/9, nsp10, nsp12 and GP3. These residues do not correlate with virulence as the A2MC2-P90 is avirulent. The nsp8 has unknown functions. The nsp9 is the RNA-dependent RNA polymerase; nsp10 is the helicase and GP3 is a structural glycoprotein^32^,^33^. The nsp12 induces STAT1 phosphorylation at Ser727 and may contribute to expression of inflammatory genes^34^. None of these genes are known to be involved in the PRRSV antagonizing feature of interferon induction. It is not known whether these proteins or the nucleotide-related RNA structures play a role in inducing interferon induction. Further studies are needed to address the question.

Compared to the wild type virus, A2MC2-P90 genome has a deletion of 543 nucleotides in nsp2. The deletion likely occurred around passage 60 as there were both 719 and 1262 bp bands in the PCR of passage 60, but the 1262 bp band disappeared in passage 61. It appears that the virus with deletion grows faster and quickly became the main virus in passage 61. A2MC2-P90 propagates faster with higher yield in MARC-145 cells than the wild type A2MC2. Our results are consistent with an earlier report that the spontaneous nsp2 deletion contributes to the faster virus propagation in a different strain *in vitro*^35^. However, the nsp2 deletion does not have an effect on PRRSV virulence for that strain *in vivo.*

In addition, A2MC2-P90 has 35 nucleotide differences compared to the wild type virus, scattered throughout the genome. Nsp9 and nsp10 were found to contribute to fatal virulence of high pathogenic PRRSV strains in China^36^. There are one and three different amino acid residues in nsp9 and nsp10, respectively, between A2MC2-P90 and the wild type virus (Table 1). Compared with moderate virulent VR-2332 and its derived avirulent MLV, these residues in the wild type A2MC2 are not unique (Supplemental Table 1), suggesting that their correlation with virulence is unlikely. There is no unique synonymous mutations in nsp9 and nsp10 in A2MC2-P90. It is thus unknown which nucleotide mutations contribute to the attenuation of A2MC2, possibly a combined effect of the multiple mutations has to be considered. Among the 35 nucleotide mutations of A2MC2-P90 in comparison with the wild type A2MC2, only nt13011 is the same as in the MLV but different from VR-2332, leading to serine in both A2MC2-P90 and the MLV, and glycine in strains A2MC2 and VR-2332. The significance of this one amino acid variation between both moderate virulent strains and their avirulent descendants is not known and may need to be investigated.

A2MC2-P90 virus replicates faster in MARC-145 cells by inducing larger plaques and having higher titer of virus yield. It appears that A2MC2-P90 virus is less sensitive to the interferons it induces, as it replicates well when the cells are inoculated at an MOI of 1, while the wild type replicates poorly at this amount of inoculum^15^. This indicates that A2MC2-P90 virus has been adapted to the cells and may gain the ability to dampen the interferon-activated antiviral response.

The animal studies demonstrate that A2MC2-P90 is avirulent and elicits better adaptive immune response than the MLV strain. The ELISA result showed that all PRRSV-infected pigs were seroconverted by DPI35. The VN test result showed most infected pigs had detectable VN antibodies by DPI28. The pigs infected with A2MC2-P90 had higher VN titers than the MLV group. The results indicate that the high passage of A2MC2 carries similar immunogenicity as the wild type virus.

In conclusion, the attenuation of A2MC2 was accomplished by serial passaging in MARC-145 cells. The unique feature of interferon induction in both MARC-145 and PAM cells sustains the 90 serial passaging. A2MC2-P90 propagates more vigorously in MARC-145 cells than wild type A2MC2. A2MC2-P90 is avirulent in pigs. Sequence analysis shows A2MC2-P90 has a 543-nucleotide deletion in nsp2 and 35 nucleotide mutations throughout the genome in comparison to the wild type virus. A2MC2-P90 is avirulent and elicits higher VN antibodies than the Ingelvac PRRS^®^ MLV strain. Further characterization of the attenuated virus is warranted for development of an improved vaccine against PRRS.

## Additional Information

**How to cite this article**: Ma, Z. *et al.* Sustaining Interferon Induction by a High-Passage Atypical Porcine Reproductive and Respiratory Syndrome Virus Strain. *Sci. Rep.* **6**, 36312; doi: 10.1038/srep36312 (2016).

**Publisher’s note:** Springer Nature remains neutral with regard to jurisdictional claims in published maps and institutional affiliations.

## Supplementary Material

Supplementary Information

## Figures and Tables

**Table 1:** List of non-synonymous nucleotide mutations and their derived amino acids in A2MC2-P90 genome compared to the wild type A2MC2^a^.

Position^b^	Nucleotide^c^		Amino acid^d^		Protein^e^
	A2MC2-P90	A2MC2	A2MC2-P90	A2MC2	
1414	G	A	A	T	nsp2/TF/N
1568	G	A	G	E	nsp2/TF/N
3706	T	C	S	P	nsp2/TF/N
5369	C	T	T	I	nsp3
6520	A	G	T	A	nsp5
7168	A	G	I	V	nsp7a
7171	C	G	H	D	nsp7a
7606	A	G	I	V	nsp8/nsp9
9729	G	A	A	T	nsp10
10122	G	A	V	I	nsp10
11197	T	A	F	Y	nsp11
12361	G	A	V	M	GP2a
12613	G	A	V	I	GP2a
13011	A	G	S	G	GP3
13264	T	C	L	S	GP3
13367	G	A	G	S	GP4
13409	A	G	N	D	GP4
13475	A	G	I	V	GP4
13798	T	A	I	K	GP5
			D	E	GP5a
14026	T	G	V	G	GP5
14344	T	C	V	A	GP5
15219	G	A	A	T	N

^a^GenBank accession numbers: A2MC2 (GenBank ID: JQ087873) and A2MC2-P90 (GenBank ID: KU318406). Some nucleotides locate in ORF overlap regions and result in different amino acids in the corresponding ORF.

^b^Nucleotide positions are indicated on the left column based on A2MC2 genomic sequence.

^c^Nucleotides at the indicated the genomic sequence positions are listed.

^d^Amino acids derived from the codon of indicated nucleotides are listed.

^e^PRRSV viral proteins corresponding to the amino acids derived from the codon of indicated nucleotide positions are listed on the right column.
