# Supplementary material for: Sustaining Interferon Induction by a High-Passage Atypical Porcine Reproductive and Respiratory Syndrome Virus Strain
Source: Sci Rep. 2016 Nov 2;6:36312. doi: 10.1038/srep36312 (PMC5090871; doi:10.1038/srep36312)
Supplement: Supplementary Information [file srep36312-s1.doc]

**Supplemental Table**

**Title: Sustaining Interferon Induction by a High-Passage Atypical Porcine Reproductive and Respiratory Syndrome Virus Strain**

Authors: Zexu Ma, Ying Yu, Yueqiang Xiao, Tanja Opriessnig, Rong Wang, Liping Yang, Yuchen Nan, Siba K. Samal, Patrick G. Halbur, and Yan-Jin Zhang

Supplemental table 1. List of nucleotide and amino acid variations in A2MC2-P90 in comparison with A2MC2, VR-2332, and IngelVac PRRS MLV strains^a^.

| Position^b^ | Nucleotide^c^ | | | | Amino acid^d^ | | | | Protein^e^ |
| --- | --- | --- | --- | --- | --- | --- | --- | --- | --- |
|  | A2MC2-P90 | A2MC2 | MLV | VR-2332 | A2MC2- P90 | A2MC2 | MLV | VR-2332 |  |
| 102 | G | A | A | A | - | - | - | - | -^f^ |
| 784 | G | G | A | G | V | V | I | V | nsp1β |
| 1027 | T | C | C | C | L | L | L | L | nsp1β |
| 1181 | C | C | T | C | S | S | F | S | nsp1β |
| 1414 | G | A | A | A | A | T | T | T | nsp2/TF/N |
| 1568 | G | A | A | A | G | E | E | E | nsp2/TF/N |
| 1998 | T | C | C | C | D | D | D | D | nsp2/TF/N |
| 2192 | C | C | T | C | S | S | F | S | nsp2/TF/N |
| 2658 | G | A | A | A | K | K | K | K | nsp2/TF/N |
| 3040 | - | G | A | G | - | D | N | D | nsp2/TF/N |
| 3433 | - | G | G | G | - | V | V | V | nsp2/TF/N |
| 3457 | - | G | A | G | - | D | N | D | nsp2/TF/N |
| 3706 | T | C | C | C | S | P | P | P | nsp2/TF/N |
| 4086 | T | C | C | C | G | G | G | G | nsp2/TF |
| 4681 | G | G | G | T | A | A | A | S | nsp3 |
| 4944 | C | C | T | T | A | A | A | A | nsp3 |
| 5097 | G | G | A | G | R | R | R | R | nsp3 |
| 5369 | C | T | T | T | T | I | I | I | nsp3 |
| 5448 | C | C | T | T | V | V | V | V | nsp3 |
| 5781 | G | A | A | A | Q | Q | Q | Q | nsp4 |
| 6345 | A | A | T | A | P | P | P | P | nsp5 |
| 6519 | G | G | G | G | A | A | A | A | nsp5 |
| 6520 | A | G | G | G | T | A | A | A | nsp5 |
| 6674 | T | T | T | C | L | L | L | P | nsp5 |
| 7168 | A | G | G | G | I | V | V | V | nsp7a |
| 7171 | C | G | G | G | H | D | D | D | nsp7a |
| 7449 | G | G | A | A | G | G | G | G | nsp7b |
| 7554 | C | C | T | T | V | V | V | V | nsp7b |
| 7606 | A | G | G | G | I | V | V | V | nsp8/nsp9 |
| 7621 | T | T | A | A | S | S | T | T | nsp8/nsp9 |
| 7754 | G | G | A | A | T | T | T | T | nsp9 |
| 9617 | A | A | G | G | E | E | E | E | nsp9 |
| 9627 | T | G | T | T | S | A | S | S | nsp10 |
| 9655 | T | T | C | C | L | L | P | P | nsp10 |
| 9729 | G | A | A | A | A | T | T | T | nsp10 |
| 9918 | T | T | C | T | L | L | L | L | nsp10 |
| 9958 | A | A | A | G | E | E | E | G | nsp10 |
| 10037 | C | C | T | T | N | N | N | N | nsp10 |
| 10122 | G | A | A | A | V | I | I | I | nsp10 |
| 10533 | T | T | C | T | Y | Y | H | Y | nsp10 |
| 10697 | C | C | C | T | A | A | A | A | nsp10 |
| 10781 | A | A | A | G | T | T | T | T | nsp10 |
| 10803 | C | C | C | T | R | R | R | C | nsp10 |
| 10895 | C | C | T | C | D | D | D | D | nsp10 |
| 11055 | A | A | A | T | T | T | T | S | nsp11 |
| 11081 | A | A | A | G | P | P | P | P | nsp11 |
| 11169 | T | C | C | C | L | L | L | L | nsp11 |
| 11197 | T | A | A | A | F | Y | Y | Y | nsp11 |
| 11221 | A | A | A | G | E | E | E | G | nsp11 |
| 11229 | G | G | T | G | V | V | L | V | nsp11 |
| 11329 | C | C | C | G | A | A | A | G | nsp11 |
| 11450 | G | A | A | A | K | K | K | K | nsp11 |
| 11666 | T | T | T | C | P | P | P | P | nsp12 |
| 11681 | A | G | G | G | V | V | V | V | nsp12 |
| 12012 | G | G | A | A | G | G | S | S | nsp12 |
| 12102 | G | G | T | G | L | L | F | L | GP2a |
|  |  |  |  |  | D | D | Y | D | GP2b |
| 12261 | T | C | C | C | A | A | A | A | GP2a |
|  |  |  |  |  | S | P | P | P | GP2b |
| 12330 | C | T | T | T | I | I | I | I | GP2a |
| 12361 | G | A | A | A | V | M | M | M | GP2a |
| 12600 | G | G | T | G | G | G | G | G | GP2a |
| 12613 | G | A | A | A | V | I | I | I | GP2a |
| 12943 | G | G | A | G | G | G | E | G | GP3 |
| 12950 | C | C | T | C | D | D | D | D | GP3 |
| 12972 | G | G | A | A | V | V | M | M | GP3 |
| 12975 | G | G | A | A | V | V | I | I | GP3 |
| 13011 | A | G | A | G | S | G | S | G | GP3 |
| 13264 | T | C | C | C | L | S | S | S | GP3 |
|  |  |  |  |  | L | L | L | L | GP4 |
| 13367 | G | A | A | A | Q | Q | Q | Q | GP3 |
|  |  |  |  |  | G | S | S | S | GP4 |
| 13409 | A | G | G | G | A | A | A | A | GP3 |
|  |  |  |  |  | N | D | D | D | GP4 |
| 13475 | A | G | G | G | I | V | V | V | GP4 |
| 13654 | T | T | C | C | V | V | V | V | GP4 |
| 13798 | T | A | A | A | I | K | K | K | GP5 |
|  |  |  |  |  | D | E | E | E | GP5a |
| 13825 | G | G | A | G | R | R | Q | R | GP5 |
|  |  |  |  |  | A | A | A | A | GP5a |
| 14026 | T | G | G | G | V | G | G | G | GP5 |
| 14238 | A | A | G | A | R | R | G | R | GP5 |
| 14344 | T | C | C | C | V | A | A | A | GP5 |
| 14420 | C | C | G | C | Q | Q | E | Q | M |
| 14446 | C | T | T | T | I | I | I | I | M |
| 14735 | G | G | G | C | G | G | G | R | M |
| 14737 | C | C | C | G | G | G | G | R | M |
| 14903 | T | T | C | C | N | N | N | N | N |
| 15219 | G | A | A | A | A | T | T | T | N |

1. GenBank accession numbers: VR-2332 (GenBank ID: U87392), IngelVac PRRS MLV (GenBank ID: AF066183), A2MC2 (GenBank ID: JQ087873) and A2MC2-P90 (GenBank ID: KU318406). Some nucleotides locate in ORF overlap regions and result in different amino acids in the corresponding ORF.
2. Nucleotide positions are indicated on the left column based on VR-2332 genomic sequence.
3. Nucleotides at the indicated genomic sequence positions are listed. The residues highlighted in blue color indicate those different from A2MC2.
4. Amino acids derived from the codon of indicated nucleotides are listed. The residues highlighted in red color indicate those different from A2MC2.
5. PRRSV viral proteins corresponding to the amino acids derived from the codon of indicated nucleotide positions are listed on the right column.
6. The nt102 locates in the 5’ UTR.
